# Infertile Women’s Opinion Concerning Gestational Surrogacy: A Systematic Review and Meta-Analysis

**DOI:** 10.18502/ijph.v49i8.3866

**Published:** 2020-08

**Authors:** Sanaz MUSAVI, Hossein MASHHADI ABDOLAHI, Morteza GHOJAZADEH, Mahdieh ABBASALIZAD FARHANGI, Zeinab NIKNIAZ, Leila NIKNIAZ

**Affiliations:** 1. Women’s Reproductive Health Research Center, Tabriz University of Medical Sciences, Tabriz, Iran; 2. Tabriz Health Services Management Research Center, Health Management and Safety Promotion Research Institute, Tabriz University of Medical Sciences, Tabriz, Iran; 3. Research Center for Evidence-Based Medicine, Health Management and Safety Promotion Research Institute, Tabriz University of Medical Sciences, Tabriz, Iran; 4. Drug Applied Research Center, Tabriz University of Medical Sciences, Tabriz, Iran; 5. Liver and Gastrointestinal Diseases Research Center, Tabriz University of Medical Sciences, Tabriz, Iran

**Keywords:** Infertility, Attitude, Gestational surrogacy, Meta-analysis

## Abstract

**Background:**

This systematic review and meta-analysis of the observational studies aimed at evaluating the infertile women’s attitude toward gestational surrogacy.

**Methods:**

Published studies until Jan 2019 were searched using PubMed/MEDLINE, Scopus, EMBASE, Cochrane Library, ISI Web of Science, Proquest and Google scholar, MagIran, SID and IranMedex. Studies in English or Persian language surveyed attitudes toward surrogacy for infertile women published until Jan 2019. Animal studies and studies with poor methodological quality were excluded from the review.

**Results:**

Six eligible studies including 1359 infertile women were identified. Of these, 559 and 742 women agreed and disagreed with surrogacy respectively. The overall event rate of positive attitude for surrogacy in infertile women was %39.7 (%95 CI=24.5 to 57.1, P=0.245).

**Conclusion:**

The infertile women’s attitude toward surrogacy is not strongly positive. We believe, more studies should be conducted among different socioeconomic, religious and cultural groups.

## Introduction

Infertility was defined as the “Inability to conceive within one year of exposure to pregnancy (i.e.-being sexually active, using non-contraception, and non-lactating) among women aged 15–49 years” (1). It is a common problem affecting one couple out of every six couples (2). The prevalence of infertility is 5% to 10% among couples worldwide (3). In vitro fertilization (IVF) techniques have been bringing confidence to infertile couples since the delivery of the first IVF baby (4). Nevertheless, a healthy female with a healthy uterus and gametes are essential to apply these procedures. If IVF is not a viable treatment option for couples, other assisted human reproduction (AHR) options such as donated gametes or surrogacy should be considered (5).

Although surrogacy does not seem to be a publicly desirable option to overcome involuntary childlessness (5). In many countries, surrogacy and its related regulations are now established. Gestational and genetic surrogacy are the two types of surrogacy proposing a full or partial genetic link. In gestational surrogacy, both the intended mother and father use their gametes and the genetically related embryo is transferred into the surrogate mother via IVF. However, in genetic surrogacy, baby is genetically linked to the surrogate mother and intended father (6).

Opinions surrounding surrogacy and infertility have been changed over time (5). Despite the plethora of debates on the psychological, moral, legal and ethical implications of surrogacy, inadequate investigations on what the public’s attitude is and its related factors has been done (5, 7–9). However, little research has been conducted to spectacle the infertile women’s opinion about the surrogacy and how satisfactory this IVF technique is to them. There is no systematic review and meta-analysis to summarize the results of this researches. Therefore, this systematic review and meta-analysis aimed at evaluating the evidence about the infertile women’s attitudes towards gestational surrogacy.

## Methods

All procedures performed in this study were in accordance with the ethical standards of the Ethics Committee of Tabriz University of Medical Science (Ethic’s number: 100.34).

### Data sources

Published studies until Jan 2019 were collected for systematic review of infertile women’s attitudes toward surrogacy by searching PubMed/MEDLINE, Scopus, EMBASE, Cochrane library, ISI Web of Science, Proquest and Google Scholar, Scientific Information Database, Magiran, and Barakat knowledge network system. Keywords were selected based on Mesh terms and included (but not limited to):“Surrogacy” OR “Surrogate” AND “Attitude” OR “Viewpoint,” OR “Belief,” OR “Opinion,” AND “Mother,” OR “Women” AND “Infertile”. The references of recent reviews and other eligible articles were manually searched for additional studies not identified by the electronic search.

Criteria for considering studies were as follow Studies in English or Persian language surveyed attitudes toward surrogacy in infertile women published until Jan 2019. Animal studies and studies with poor methodological quality were excluded from the review.

### Study Selection, Data Extraction Strategy, Quality Assessment

Two independent reviewers screened all titles and abstracts of the retrieved papers to decide on their inclusion. In addition, full articles of the potentially relevant studies were retrieved and independently screened for eligibility by two reviewers. Disagreements were resolved through discussion, and the reasons for exclusion were recorded for each of the excluded full-text articles. Two researchers also conducted data extraction independently, with disagreements being resolved through discussion.

For data extraction, a manual data extraction form was designed in the Microsoft Word environment. At first, data from three papers were extracted experimentally for these forms, and the deficiencies and problems encountered in the initial form were resolved. For each eligible study, data on the study method, population characteristics, and results were extracted by one reviewer and checked by a second reviewer. Any discrepancies were resolved through discussion and by consulting a third reviewer.

### Quality assessment

Two Reviewers assessed the methodological quality of selected studies using Joanna Briggs Institute (JBI) Critical Appraisal Checklist for prevalence Studies (inter-rater reliability of 0.98) (10).

### Quantitative synthesis

All analyses were carried out using the Comprehensive Meta-Analysis version 2.0. We extracted mean (range) age of the participants, duration of marriage, number of items and reliability of questionnaires, the percentage and number of people they agree and disagree with surrogacy and then the event rates were computed.

### Statistical Analysis

Heterogeneity was determined using the Q statistic and I^2^. A significance level of *P*<0.10 for Cochran’s Q test or I^2^>50% was considered as clinically important heterogeneity (11). Publication bias was assessed graphically using a funnel plot and mathematically using an adjusted rank correlation test, according to methods of Begg and Mazumdar and Eggers. The number of agreed attitudes and number of samples extracted from each study was used to obtain the pooled event rate.

### Search results and study characteristics

**Fig. 1:**
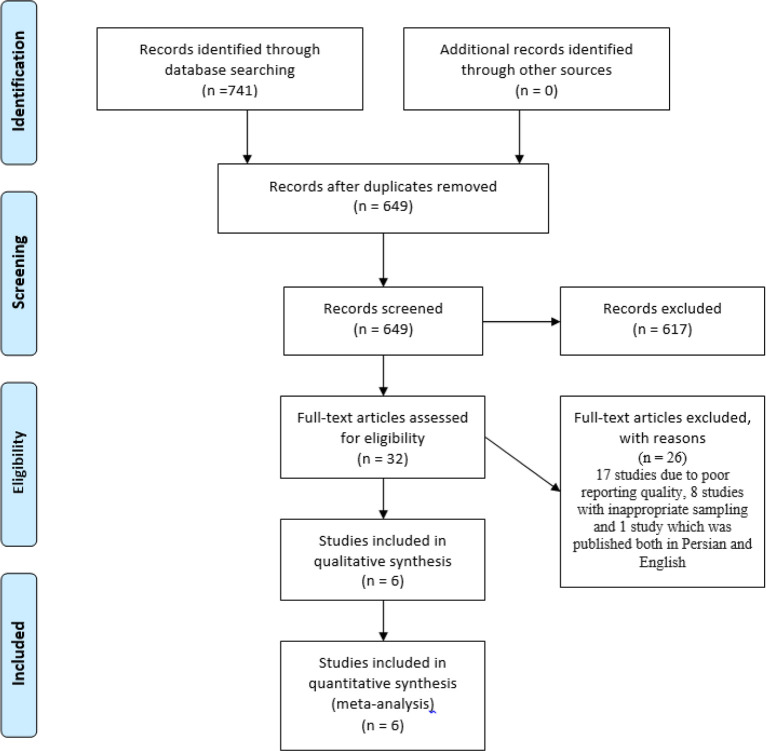
Flow diagram for selection of articles

The search strategy resulted in 741 titles, reduced to 649 following deletion of duplicates using the Endnote 7.2.1 literature manager software. Initial assessment of the titles and abstracts reduced the number of papers to 32 and further reduced to 6 on closer assessment of the full text based on the inclusion and exclusion criteria. A flow chart of literature retrieval is shown in Fig. 1. The basic characteristics of included studies are summarized in Table 1 (12–17).

**Table 1:** summary of the results of studies assessing attitudes of infertile women towards surrogacy

***Author***	***Country***	***Sample size***	***Age Range/Mean***	***Duration of marriage years***	***Questionnaire***		***Attitude N (%)***	

					***Items***	***Reliability***	***Agree***	***Disagree***
Baykal B (2008)	Turkey	368	30.23	7.23	-	-	54(14.67)	303(82.33)
Saito Y & Matsuo H (2009)	Japan	103	25–43	5.4	-	-	18(17.47)	78(75.72)
Ahmari TH. et al. (2012)	Iran	300	22–43	NM	16	0.92	167(61.3)	133(38.7)
Kazemifard Y. et al. (2013)	Iran	200	21–69	13.48	17	0.72	103(51.6)	57(28.4)
Kian ME. et al. (2014)	Iran	150	17–45	3.4	17	0.89	80(53.3)	70(46.6)
Darghahi G. et al. (2014)	Iran	238	26.9	5.3	22	0.89	137(57.8)	101(42.2)

### Participant characteristics

Of included studies, 4 were conducted in Iran (14–17), one in Japan (13) and one in Turkey (12). The years during which data were collected ranged between 2008 and 2014. In selected studies, 1359 infertile women were enrolled. Of these, 559 and 742 women agreed and disagreed with surrogacy respectively. The mean age of participants was reported in two studies (12, 17) and in others, the range of age has been reported between 17–69 years. In the studies reported the average length of marriage was 5.65 years.

### Quality of studies

All studies had one or more domains characterized as high risk of bias (Table 2). In terms of risk of selection bias, three studies were categorized as having moderate risk of selection bias (12, 13, 17). These studies did not include sampling methods. In terms of risk of outcome measurement, two studies did not mention the validity of the questionnaire used for measuring attitude (12, 13).

**Table 2:** Quality assessment of included studies using JBI Critical Appraisal Checklist for prevalence studies

***Study***	***Was the sample frame appropriate to address the target population?***	***Were study participants sampled properly?***	***Was the sample size adequate?***	***Were the study subjects and the setting described in detail?***	***Was the data analysis conducted with sufficient coverage of the identified sample?***	***Were valid methods used for the identification of the condition?***	***Was the condition measured in a standard, reliable way for all participants?***	***Was there appropriate statistical analysis?***	***Was the response rate adequate?***
Baykal B (2008)	Y	U	Y	Y	Y	U	Y	Y	Y
Saito Y & Matsuo H (2009)	Y	U	N	Y	Y	U	Y	Y	Y
Ahmari TH. et al. (2012)	Y	Y	Y	Y	Y	Y	Y	Y	Y
Kazemifard Y. et al. (2013)	Y	Y	Y	Y	Y	Y	Y	Y	Y
Kian ME. et al. (2014)	Y	Y	Y	Y	Y	Y	Y	Y	Y
Darghahi G. et al. (2014)	Y	U	Y	Y	Y	Y	Y	Y	Y

Y= Yes

N= No

U= Unclear

### Meta-analysis results

**Fig. 2:**
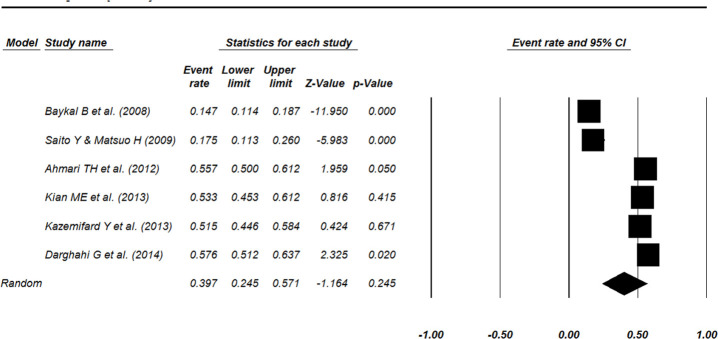
Forest plot showing the weighted event rate for women’s opinion concerning gestational surrogacy. Studies are represented as squares and the area of the square represents the weight given to the study in meta-analysis by CMA software. The overall event rate was calculated by a random-effects model. The diamond represents the overall estimated event rate and its 95% CI

According to identification of statistical heterogeneity (chi-square=175.133, *P*<0.0001 and I^2^=97.14%), the random effect model was used. The overall event rate of positive attitude for surrogacy in infertile women was %39.7 (%95 CI=24.5 to 57.1, *P*=0.245) (Fig. 2).

## Discussion

Infertile couples struggled for years to have a child may benefit from third-party reproduction method to be families with children. The opinion of infertile women toward gestational surrogacy has been investigated in six observational studies. These studies comprised of 1359 infertile women. Of included studies, 4 were conducted in Iran. About 39.7% of the infertile women have positive attitude toward surrogacy. The positive view of surrogacy in studies conducted in Iran was higher than the other studies conducted in Turkey (12) and Japan (13). Maternal marriage duration and age are main issues affecting women’s approach toward ART. In the included studies, these two factors are almost similar in all of the studies. Other factors such as culture, religion and social status seem to be significant concerns affecting infertile women’s view concerning gestational surrogacy (12). The procedure of having an assisted reproduction technology baby is decided by society through legislation (18). The legislation, without the training of society, prohibits various significant reproductive treatments. Based on the Turkish law implementation act on assisted reproduction treatment, all third party reproduction is severely forbidden (12). As well, in Japan, a legal system for the regulation of ART has not been proven (19). The rules set by the Japan Society of Obstetrics and Gynecology support donor insemination but not surrogacy (19). Surrogacy is also not permitted in Australia (South and West), Poland, Saudi Arabia, Denmark, Egypt, France, Germany, Spain, Switzerland Ireland, Italy, Sweden, Jordan, Norway, Austria, the Czech Republic, Singapore, and Taiwan (20). On the contrary, in Iran, surrogacy is not forbidden religiously and it has been practiced with no resistance (21). Legal authorization of surrogacy complemented by individual beliefs and knowledge about the method lead to its social approval. On the other hand, legislation needs to reflect the desires of society. Hence, legislative changes that identify the commissioning parents as the child’s legal parents would permit the practice of surrogacy to carry on and possibly flourish in the world. Reproductive choice is essentially a decision made by an infertile couple, but the procedure of having an assisted reproduction technology baby is decided by culture and society through legislation (18).

The other factor may causes higher acceptance rate of surrogacy in some countries is that infertile women experience more psychological turmoil due to both the social pressure to be a mother and fear of losing marriage. This condition resulted in loss of women’s self-esteem; subsequently, they seek more evidences about fertility, start the handling of the problem as soon as possible, and become more hopeful of having a child (8).

In addition, further efforts are required to increase knowledge and understanding about surrogacy. This would be necessary not only at the level of educating infertile couples but also at the level of the overall population. This would form a general understanding – and approval – of surrogacy as an appropriate technique not only to be considered and used by infertile couples but also to have willing fertile females to act as surrogate mothers (14).

### Strengths and Limitations

Our analysis has some limitations. First, publication bias cannot be excluded, i.e., negative findings are less likely to be published. Second, any systematic review is only as good as the included studies. Some of the studies included in our systematic review had potential limitations such as small sample size and applying different questionnaires for evaluating attitude. Moreover, most of the studies were conducted in one country which could distort the results.

## Conclusion

The systematic review and meta-analysis of the observational studies included in the present study indicated that the acceptance rate of using gestational surrogacy is low. Policymakers and decision-makers must review and revise the regulations of third party reproduction as infertile couples deserve the right to attempt any treatments that are available for their condition.

## Ethical considerations

Ethical issues (Including plagiarism, informed consent, misconduct, data fabrication and/or falsification, double publication and/or submission, redundancy, etc.) have been completely observed by the authors.
